# Problematic Internet Use and Smoking among Chinese Junior Secondary Students: The Mediating Role of Depressive Symptomatology and Family Support

**DOI:** 10.3390/ijerph16245053

**Published:** 2019-12-11

**Authors:** Phoenix Kit-han Mo, Ji-Bin Li, Hui Jiang, Joseph T. F. Lau

**Affiliations:** 1 Center for Health Behaviours Research, School of Public Health and Primary Care, The Chinese University of Hong Kong, Hong Kong, China; phoenix.mo@cuhk.edu.hk (P.K.-h.M.); 1155102419@link.cuhk.edu.hk (H.J.); 2 The Chinese University of Hong Kong Shenzhen Research Institute, Shenzhen 518057, China; 3 Department of Clinical Research, State Key Laboratory of Oncology in South China, Collaborative Innovation Center for Cancer Medicine, Sun Yat-sen University Cancer Center, Guangzhou 510060, China; lijib@sysucc.org.cn

**Keywords:** internet addiction, social networking addiction, smoking, depression, family support, adolescents

## Abstract

*Background*: Internet use is significant public health issue and can be a risk factor for other addictive behaviors, such as smoking. The present study examined the association between problematic Internet use (PIU) (i.e., Internet addiction (IA) and social networking addiction (SNA)) and smoking, and the mediating role of depressive symptomatology and family support played in such associations. *Methods*: A cross-sectional study was conducted among 5182 junior secondary students (grade 7 and 8) recruited from nine schools using stratified sampling. *Results*: A total of 3.6% of students had smoked in the past month, and 6.4% of students were identified as IA cases. Adjusted for significant background variables, PIU (ORa = 2.07, 95% CI = 1.48, 2.90 for IA, ORa = 1.26, 95% CI = 1.09, 1.47 for SNA), and probable depression (ORa = 1.33, 95% CI = 1.05, 1.69) were significant risk factors, while family support (ORa = 0.85, 95% CI = 0.77, 0.94) was a significant protective factor of smoking. The mediation effects of lower family support and probable depression on the association between score on IA scale and smoking, and the mediation effect of lower family support on the association between score on SNA scale and smoking were significant, while the mediation effect of probable depression on the association between score on SNA scale and smoking was marginally significant. *Conclusions*: PIU contributed to an increased risk of smoking through depressive symptomatology and decreasing family support among junior school students. Interventions to reduce smoking are warranted; they should seek to reduce problematic Internet use and depressive symptomatology, and promote family support.

## 1. Introduction

Smoking is considered as one of the leading preventable causes of death in the world [1] and is largely initiated and established during adolescence [2]. In China, a lower age of smoking onset has been observed and smoking among adolescents has been commonly reported [3]. One study among vocational high school students found that 45% had initiated smoking and 25% smoked in the past month [4]. Another study among junior high school students has shown that 5.6% have ever smoked in the past month [5]. Starting to smoke in adolescence predicts future smoking patterns [6,7] and also other problem behaviours such as substance use, violent behaviours, dropping out of school, and risky sexual behaviours [8,9]. Adolescent smoking has also been found to be associated with various negative health outcomes, such as sleeping disorders [10] and respiratory infections [11]. It is therefore important to identify factors associated with smoking among adolescents so that effective interventions could be designed. 

Factors related to adolescent smoking have been extensively examined and documented. These included background characteristics such as lower socio-economic status [12,13], cognitive factors such as positive attitudes or less negative attitudes towards smoking [5,13], psychological factors such as stress, anxiety and depression [14,15,16], lower level of self-esteem [13,14], and interpersonal factors such as peer influences [17,18,19] or low level of parental support [20,21]. 

With the rapid growth of the Internet and the social media, the emerging issue of problematic Internet use (PIU) might pose additional challenges to the smoking concern among adolescents. A meta-analysis of 31 nations across seven world regions showed that a global prevalence of IA was estimated at 6.0% [22]. In China, one study among 1173 Chinese college students found that 15.2% were classified as having Internet addiction (IA) [23]; another study have reported that 12% of young smart-phone users (mean age = 26) were classified as probable problematic social networking use [24]. 

As smoking and PIU are both considered addictive behaviors, it might be possible that they share similar underlying roots. It has been shown that that both Internet gaming disorder and nicotine dependence share similar neural mechanisms related to craving and impulsive inhibitions [25]. Similarly, a few studies have documented the association between PIU and smoking. For example, one study investigated the association of smoking and PIU among adolescents reported a dose-dependent relationship between smoking and IA, in which IA tended to increase with current smoking habit or the number of cigarette smoked [26]; a longitudinal study among high school students in China and USA reported a positive predictive relationship between baseline compulsive Internet use and change in substance use (which included smoking) at one-year follow-up among female students [27]. 

Despite the numerous studies that have reported an association between PIU and smoking, very few studies have explored the mechanisms which PIU might lead to smoking. In the present study, we hypothesized that lower level of support from family and higher level of depressive symptomatology might be the potential mediating factors between PIU and smoking. There is significant concern that adolescents who are frequently exposed to the Internet may substitute the Internet for direct human interactions, leading to decreased level of social support and increased level of depression. Indeed, studies have shown that depression is one of the most common comorbidity of PIU across various populations [28,29]. For instance, a longitudinal study among Chinese adolescents showed that those who have developed IA exhibited increased depression more than that non-addiction group at the one-year follow up [30]. 

PIU has also found to be significantly associated with parental conflict, social isolation, and low level of social support in various studies [28,29]. One longitudinal study has found that greater use of the Internet was associated with declines in participants’ communication with family members in the household, declines in the size of their social circle, and increases in their depression [31]. In China, studies have shown that students who reported poorer parent–child relationships, higher levels of depression, and lower levels of psychosocial competence were more likely to report behaviors indicative of IA [23]. Alternatively, the association between lower level of family support and higher level of depression with smoking has been widely documented [15,16,20,21,32]. 

Despite the extensive evidence on the association between PIU, family support, and depressive symptomatology, and between family support, depressive symptomatology, and smoking, no studies to date have examined the mediating role of these psychosocial variables on the relationship between PIU and smoking. In addition, most of the evidence reported to date have mainly focused on IA, while relatively less studies have been conducted on the association between social networking addiction (SNA), family support, depressive symptomatology, and smoking. The present study examined the prevalence of smoking, and the association between PIU (as measured by IA and SNA) and smoking among adolescents in China. The potential mediators, including depressive symptomatology and lower level of family support, were also examined. 

## 2. Materials and Methods

### 2.1. Target Participants

Participants were grade 7–8 students from nine public junior middle schools in Guangzhou, Mainland China. Grade nine students were excluded as they would need to prepare for the high school entrance examination. 

### 2.2. Sampling

A stratified cluster sampling method was applied to recruit participants. First, one district was selected from each of the three regions (core region, suburb region and outer suburb region) of Guangzhou, respectively using convenience sampling. Second, three junior middle schools were selected from each selected district/county using convenience sampling. A total of nine schools were thus selected. All grade 7 and 8 students from the selected schools were invited to take part in the study. 

### 2.3. Participant Recruitment

Consent and permission to administer the survey was obtained by school principals prior to data collection. An anonymous structured questionnaire was administered to the students by trained field workers in the absence of teachers in classroom settings. Information on the study’s background and purpose was printed in the cover page of the questionnaire. The voluntary nature and the right of refusal to take part in the study were also highlighted by the field workers. Participants received no incentive and return of the questionnaire implied informed consent. The study was approved by the Survey and Behavioral Research Ethics Committee of the Chinese University of Hong Kong.

A total of 5472 students completed the survey. Only participants who have provided complete data on the studied variables were included for analysis. As a result of this, 290 participants were excluded from the analysis, resulting in a total of 5182 valid responses. 

### 2.4. Measures

Background characteristics. Participants were asked about their gender, grade, and parental educational attainment. They were also asked whether they were social networking users, and to rate their own academic pressure and perceived academic performance. 

PIU. PIUs were measured by scores of IA and SNA. IA was measured by the eight-item Young’s diagnostic questionnaire (YDQ) [33]. All items involved “yes/no” response categories and participants who provided five or more “yes” answers were classified as cases of IA [34]. The scale has commonly been used in the Chinese student population [35,36]. The Cronbach’s *α* of the scale was 0.66 in the present study. SNA was measured by a modified version of the Facebook addiction scale [37], which included eight items describing addictive symptoms (i.e., cognitive and behavioral salience, conflict with other activities, euphoria, loss of control, withdrawal, and relapse and reinstatement). In the present study, the word “Facebook” was replaced by “online social networking”, and translation and back-translation processes were implemented by two bilingual researchers. Response categories rated from 1 = not true to 5 = extremely true. The total score of the scale ranged from 8 to 40, with a higher score indicating a higher level of addictive tendency to social networking. The scale showed good internal reliability in the present study (Cronbach’s *α* = 0.86).

Depressive symptomatology. Depressive symptomatology was assessed using the Chinese version of the 20-item Center for Epidemiological Studies-depression scale (CES-D) [38]. The CES-D is one of the most commonly used self-report instruments in screening depressive symptomatology [39]. It has been used in Chinese adolescent population [28,40]. All items were responded on a four-point Likert scale ranging from 0 = rarely or none of the time (less than 1 day) to 3 = almost or all of the time (5–7 days). The total score ranged from 0 to 60, with a higher score reflecting more depressive symptoms. As suggested by the scale, a score of 16 to 20 was classified as mild depression, 21 to 24 was classified as moderate depression, and 25 or above was classified as severe depression [40]. Based on the suggested cut-off, participants who scored higher than 16 were classified as probable depression in the present study. The scale showed good internal reliability in the present study (Cronbach’s *α* = 0.86).

Family support. Family support was measured by the four-item perceived family support subscale of the multidimensional scale of perceived social support (MSPSS) [41]. It has been used on the Chinese adolescent population [42]. Each item was rated on a seven-point Likert scale ranging from 1 = very strongly disagree to 7 = very strongly agree, with higher scores indicating higher level of perceived family support. The scale showed good internal reliability in the present study (Cronbach’s *α* = 0.90).

Smoking. Participants were asked to report whether they have smoked in the past month, and the number of cigarettes they smoked per day. 

### 2.5. Data Analysis

Descriptive statistics (e.g., means (M), standard deviation (SD), percentages) were presented. Associations between background characteristics (i.e., socio-demographic variables and variables related to academic issues) and smoking were tested by multilevel logistic regression, with individual students as level 1 and school as level 2; their univariate odds ratios (ORu) and their respective 95% confidence interval (CI) were derived. For IA and depressive symptomology, binary variable was created based on the suggested cut-off. For the continuous variables (i.e., SNA and family support), We standardized the score by its mean and standard deviation to calculate the Z-scores respectively. Multiple multilevel logistic regression models were then fit separately for each of the PIU (i.e., score on IA/SNA scale) and psychosocial (i.e., family support, probable depression) variables on smoking, adjusted for background variables that were significant at the *p* < 0.05. Resulting adjusted odds ratio (ORa) and 95% CI were reported. 

Next, based on the procedures proposed by Baron and Kenny, a series of multilevel logistic regressions were conducted to test the mediating role of depressive symptomatology and family support on the association between PIU and smoking. First, the association between the independent variable (i.e., score on IA/SNA scale) and dependent variable (i.e., smoking) was tested after adjusting for significantly background characteristics. Second, the association between the independent variable (i.e., score on IA/SNA scale) and the potential mediators (i.e., family support, probable depression) were examined, adjusting for significant background characteristics. The association between score on IA/SNA scale and family support was calculated using linear regressions (with the Z-score of family support as dependent variable); while the association between IA/SNA and probable depression was calculated using logistic regressions. Third, the independent variable (i.e., the score on the IA/SNA scale) and the potential mediators (i.e., family support, probable depression) were entered into the same model to predict smoking, adjusting for significant background characteristics. The Sobel test was performed to calculate the significance of the mediation, and the proportion of the mediation was calculated based on the procedure set out by Vanderweele et al [43]. 

## 3. Results

### 3.1. Descriptive Statistics

Slightly less than half of the participants were in 7th grade (48.0%), and similar proportion of the participants were female (47.6%). Respectively, 55.4% and 49.2% of the participants reported that their father and mother had senior secondary school level of education of above. About half (47.0%) reported that their family financial situation was very good. In terms of academic-related variables, One-third (34.2%) reported that they had an upper academic performance; and 80.6% perceived they had moderate level of study pressure or above. Most of the participants (92.2%) were social networking users, and 6.4% and 40.5% of the participants scored above the cut-off for IA and probable depression respectively. A total of 184 (3.6%) participants reported they have smoked in the past month, among them 18.5% smoked three cigarettes or more per day (Table 1).

### 3.2. Association between Background Characteristics, PIU, Psychosocial Variables and Smoking

Among the background variables, male gender (ORu = 1.47, 95% CI = 1.16, 1.87), having poor or very poor family financial situation (ORu = 2.17, 95% CI = 1.58, 2.96), and having lower level of academic performance (ORu = 1.76, 95% CI = 1.24, 2.50) were positively associated with smoking. These factors were adjusted for in subsequent analyses (Table 2).

Adjusted for significant background variables, both scores on IA and SNA scale (ORa = 2.07, 95% CI = 1.48, 2.90 for IA and ORa = 1.26, 95% CI = 1.09, 1.47 for SNA) were positively associated with smoking. Furthermore, probable depression (ORa = 1.33, 95% CI = 1.05, 1.69) was identified as a significant risk factor while family support was identified as a significant protective factor (ORa = 0.85, 95% CI = 0.77, 0.94) for smoking (Table 2).

### 3.3. Testing the Mediation Effects

Adjusting for significant background variables, both scores on IA and SNA scale were significantly associated with probable depression (OR = 4.06, 95% CI = 3.16, 5.23 for IA and OR = 1.86, 95% CI = 1.74, 1.98 for SNA) and family support (*β* = −0.41, 95% CI = −0.52, −0.30 for IA and *β* = −0.17, 95% CI = −0.20, −0.14 for SNA) (Table 3). Therefore, the requirements for testing mediation effects were fulfilled. The mediation hypothesis was then tested by entering each of the two potential mediators (i.e., family support and probable depression) individually into the regression model plus score on IA/SNA scale (Model 2 and 3 for IA and Model 5 and 6 for SNA, Table 4). Each of these models contained a single mediator plus score on IA/SNA scale, and was compared against the model of score on IA/SNA scale alone (Model 1 for IA and Model 4 for SNA, Table 4). The odds ratio of score on IA/SNA scale diminished though remained significant. For the model of association between SNA and smoking, the odds ratio of probable depression on smoking become marginally significant (Model 6, Table 4). Results of the Sobel test revealed that the mediation effects of family support and probable depression on the association between score on IA scale and smoking, and the mediation effect of family support on the association between score on SNA scale and smoking were significant (*p* < 0.05, proportion of mediation ranged from 11% to 43%, Table 5), while the mediation effect of probable depression on the association between score on SNA scale and smoking was marginally significant (*p* = 0.07, proportion of mediation 36%, Table 5). 

## 4. Discussion

Both PIU and smoking are important public health concerns among adolescents. The present study examined the association between PIU and smoking, and elucidated the mechanism underlying such an association. It is first important to point out that 3.6% of our sampled students had smoked over the past month. The prevalence was similar to those reported among junior secondary school students in China [5]. 

Our findings show that PIU is a potential significant risk factor of smoking. Results corroborate with those previous studies showing a significant association between IA and smoking [26,27] and extend the knowledge that smoking is also associated with SNA. Addiction with the Internet may interfere with normal, adaptive functioning, leading to engagement in risky behaviors. It is important to note that nearly all participants in the present study were social networking users and about 6.5% of them had IA. The figures are alarming given that the sampled students are at a very young age. Findings highlight the importance of investigating the effect of addictions to specific Internet platform (i.e., social network) and the need to designing interventions to reduce PIU, which might possibly reduce the risk of smoking among adolescents. 

The present study also suggests that 40.5% of the sample scored above the cut-off for probable depression. Using the same scale (CES-D) with the same cut-off, the prevalence of probable depression was lower than those reported among secondary school students in Hong Kong (53.2% among male and 62.1% among female) [28]. In the present study, probable depression was also shown to be a potential significant risk factor to smoking. Findings support the literature that depression is associated with an increased frequency of smoking [44,45,46], and that depressive symptomatology is more common among smokers [45]. The self-medication hypothesis, which individuals with depression might use smoking as an emotion regulation strategy to cope with their depressive mood or reduce their negative affect, might explain such association [47]. Alternatively, depressive symptomatology may leave adolescents more vulnerable to peer smoking influences, which increase their chance of smoking [47,48]. Findings suggest that improving adolescents’ mental state may be a useful strategy for smoking cessation for adolescents. 

To effectively combat adolescent smoking, it is important to promote protective factors and the same time, reduce the risk factors associated with smoking. Our findings revealed that family support was a potential protective factor to smoking, results which are consistent with the extant literature [49,50]. Adolescence represents an important period when young people strive for independence in order to establish their self-identify. During this period, family remains an important source of support and influence on their development. Support from parents is important and consequential for them in coping with stressors, and establishing a positive personal development. Adolescents with higher level of parental support might have better problem solving skills such that they would be less likely to turn to maladaptive behaviors (such as smoking) when they encounter adversities. 

Currently there is a dearth of studies looking at the mechanism between PIU and smoking. Examining the association between PIU and smoking and its underlying mechanism would help us elucidate specific factors for PIU and smoking. Such information would be useful for health care professionals to design appropriate interventions to reduce smoking among the adolescents. Findings suggest that PIU was associated with lower level of family support and higher levels of depressive symptomatology, which in turn, associated with a higher likelihood to smoke. Adolescents with PIU might prefer to turn to the Internet for communication, which ultimately lead to decreased time spend in face-to-face interactions, decreased level of support from family and increased level of depressive symptoms, and subsequently increased chance of smoking. PIU might also weaken the protective effect of family support, which increased one’s vulnerability to smoke. Interventions for smoking should not only reduce PIU but promote family support and reduce depressive symptomatology in order to lower the potential negative impact of PIU on smoking. 

### 4.1. Implications for Practice

Given the prevalence of PIU and its potential positive association with smoking, evidence-based interventions to reduce smoking are warranted. Findings of the present study suggest that reducing PIU could possibly be a useful way to combat adolescent smoking problem. A few evidence-based interventions to reduce PIU among the adolescences have been reported in the literature. For example, it has been shown that school-based group using cognitive behavioral theory (CBT) was effective in reducing IA [51]. Other studies also reported that reality therapy counselling [52], reality therapy combined with mindfulness mediation [53], and motivational interviewing [54,55] were effective in reducing PIU. Findings also suggest that reducing participants’ depressive symptoms may be a useful strategy for reducing smoking. Indeed, evidence has suggested that psychological interventions are effective in reducing smoking [56]. To develop effective interventions for reducing smoking, it is important to enhance protective factors of smoking, besides reducing risk factors. Findings of the present study suggest that interventions to increase family support may have the potential to reduce smoking. There is a need for health care professionals to understand the context of family support and the way in which adolescents obtain support from the family. Family-based approaches that focuses on improving the parent–child relationship, increasing communication and understanding among family members can be a direction for increasing family support of adolescents. Review studies have suggested that family-based interventions were effective in reducing the onset of smoking among children and adolescents [57].

### 4.2. Limitations of the Study

There are several limitations of the study that should be noted. First, the study is limited by its cross-sectional nature, therefore the association between PIU, depressive symptomatology, lower levels of family support and smoking are only suggestive. It might also be plausible that smoking leads to depressive symptomatology and lower levels of family support, which in turn lead to PIU. Longitudinal studies are warranted to elucidate the prospective relationship between the variables. Second, all data were self-reported so the levels of IA and smoking might have been underestimated. Third, due to the lack of an established cut-off point for SNA, the extent of SNA of the sample was unclear. Fourth, the association between PIU and smoking could have been mediated by other individual factors, such as individual differences in circadian rhythm [58]. Future studies should explore other potential mediators of such association. Fifth, written consent was not obtained from the students, and their return of the questionnaire implied informed consent. We have been advised by the schools that written consent was not necessary, but students needed to be briefed very thoroughly and the participation should be completely voluntary. We have taken careful steps to make sure that a very detailed briefing was provided to ensure confidentiality, anonymity, and voluntariness. Sixth, the internal reliability of YDQ was low in the present study. We were aware that other validated scales for IA (e.g., the Internet addiction test, IAT [59]) were available but due to concern about length of the questionnaire, YDQ was chosen over other validated scales. Finally, the current sample was collected from nine secondary schools in Guangzhou, generalization to other secondary school students might be limited. 

## 5. Conclusions

To conclude, findings of the present study call for a need to reduce PIU and smoking among junior secondary school students in China and provide insights on the variables that could be targeted when designing anti-smoking interventions targeting this population. In particular, findings show that PIU was associated with smoking among junior secondary school students in China and such associations were mediated by depressive symptomatology and lower levels of family support. Results provide important implications that interventions to reduce smoking among the current sample should target not only to reduce PIU, but also to reduce depression and promote family support. 

## Figures and Tables

**Table 1:** Descriptive characteristics of participants (*N* = 5182).

Variable	No. (%)/M (SD)
**Depression**	
Score of depression (Center for Epidemiological Studies-Depression Scale, CES-D)	15.17 (9.61)
Probable depression	
CES-D < 16	3085 (59.5)
CES-D 16–20	808 (15.6)
CES-D 21–24	497 (9.6)
CES-D 25 or above	792 (15.3)
**Variables related to problematic Internet use**	
Social networking user	4779 (92.2)
Score of internet addiction (IA, Young’s diagnostic questionnaire)	1.53 (1.65)
Prevalence of IA	332 (6.4)
Score of social network addiction (SNA, modified Facebook addiction scale)	16.85 (5.87)
**Variables related to smoking**	
Have smoked in the past month	
Yes	184 (3.6)
No	4998 (96.4)
Number of cigarettes smoked per day (among those who have smoked in the past month, *N* = 184)	
<1	117 (63.6)
1–2	33 (17.9)
3–4	9 (4.9)
>4	25 (13.6)

**Table 2:** Association between background characteristics, IA/SNA, psychosocial variables and smoking.

Independent Variable	Odds Ratio (Oru) (95% CI)	ORa (95% CI) †
**Socio-demographic background**		
Grade		
7th	1	−
8th	1.06 (0.86, 1.32)	−
Gender		
Female	1	−
Male	1.47 (1.16, 1.87) **	−
Father education level		
Primary school or below	1	−
Junior secondary school	0.90 (0.63, 1.30)	−
Senior secondary school	0.71 (0.47, 1.09)	−
College or above	0.70 (0.49, 1.02)	−
Do not know	0.80 (0.40, 1.58)	−
Mother education level		
Primary school or below	1	−
Junior secondary school	1.01 (0.81, 1.26)	−
Senior secondary school	1.10 (0.83, 1.46)	−
College or above	0.98 (0.80, 1.21)	−
Do not know	1.04 (0.65, 1.65)	−
Family financial situation		
Very good/good	1	−
Average	0.99 (0.91, 1.09)	−
Poor/very poor	2.17 (1.58, 2.96) ***	−
**Academic issues**		
Academic performance		
Upper	1	−
Medium	1.20 (0.98, 1.47)	−
Lower	1.76 (1.24, 2.50) **	−
Perceived study pressure		
Nil/mild	1	−
Moderate	0.86 (0.66, 1.11)	−
Heavy/very heavy	1.24 (0.87, 1.76)	−
**Variables related to problematic Internet use**		
IA		
No	1	
Yes	2.30 (1.63, 3.24) ***	2.07 (1.48, 2.90) ***
Z-score of SNA	1.31 (1.12, 1.54) **	1.26 (1.09, 1.47) **
**Psychosocial variables**		
Z-score of family support	0.83 (0.75, 0.90) ***	0.85 (0.77, 0.94) **
Probable depression (CES-D ≥ 16)		
No	1	
Yes	1.38 (1.11, 1.73) **	1.33 (1.05, 1.69) *

* *p* < 0.05, ** *p* < 0.01, *** *p* < 0.001. †: Odds ratio obtained by two-level multilevel logistic regression (level 1: student; level 2: school) adjusted by gender, family financial situation and academic performance.

**Table 3:** Association between problematic Internet use (PIU), family support and probable depression.

Independent Variable	Z-Score on Family Support	Probable Depression (CES-D ≥ 16)
	*β* (95% CI) †	OR (95% CI) §
IA	−0.41 (−0.52, −0.30) ***	4.06 (3.16, 5.23) ***
Z-score of SNA	−0.17 (−0.20, −0.14) ***	1.86 (1.74, 1.98) ***

*** *p* < 0.001; †: Obtained by two-level linear regression (level 1: student; level 2: school) adjusted by gender, family financial situation and academic performance; §: obtained by two-level logistic regression (level 1: student; level 2: school) adjusted by gender, family financial situation and academic performance.

**Table 4:** Testing mediation effect of family support and probable depression on the association between PIU and smoking.

**Independent Variable**	**Model 1**	**Model 2**	**Model 3**
	**ORa (95% CI) †**	**ORa (95% CI) †**	**ORa (95% CI) †**
IA	2.07 (1.48, 2.90) ***	1.96 (1.46, 2.64) ***	1.93 (1.44, 2.59) ***
Z-score of family support		0.87 (0.80, 0.95) **	
Probable depression			1.25 (1.01, 1.55) *
**Independent Variable**	**Model 4**	**Model 5**	**Model 6**
	**ORa (95% CI) †**	**ORa (95% CI) †**	**ORa (95% CI) †**
Z-score of SNA	1.26 (1.09, 1.47) **	1.24 (1.07, 1.44) **	1.23 (1.07, 1.43) **
Z-score of family support		0.89 (0.81, 0.97) ^*^	
Probable depression			1.18 (0.99, 1.42)

* *p* < 0.05, ** *p* < 0.01, *** *p* < 0.001 †: Odds ratio obtained by two-level multilevel logistic regression (level 1: student; level 2: school) adjusted by gender, family financial situation and academic performance.

**Table 5:** Coefficients, significance and proportion of mediation effect of family support and probable depression on the association between PIU and smoking.

Independent Variable	a	*SE*a	b	*SE*b	c’	Sobel	*p*	Proportion
**IA**								
Z-score of family support	−0.414	0.056	−0.140	0.046	0.674	2.81	0.005	0.11
Probable depression	1.402	0.129	0.224	0.108	0.656	2.04	0.041	0.43
**SNA**								
Z-score of family support	−0.171	0.015	−0.118	0.046	0.215	2.50	0.01	0.10
Probable depression	0.618	0.034	0.167	0.092	0.210	1.81	0.07	0.36

a: regression coefficient between independent variable (i.e., IA, Z-score of SNA) and the mediator (i.e., Z-score of family support, probable depression). b: coefficient between the mediator (i.e., Z-score of family support, probable depression) and the dependent variable (i.e., smoking) when both mediator and independent variable were included in the same model. c’: coefficient between independent variable (i.e., IA, Z-score of SNA) and the dependent variable (i.e., smoking) when both mediator and independent variable were included in the same model. *SE*a, *SE*b: standard errors of the corresponding coefficients.
